# Efficiencies of Internet-Based Digital and Paper-Based Scientific Surveys and the Estimated Costs and Time for Different-Sized Cohorts

**DOI:** 10.1371/journal.pone.0108441

**Published:** 2014-10-14

**Authors:** Constantin E. Uhlig, Berthold Seitz, Nicole Eter, Julia Promesberger, Holger Busse

**Affiliations:** 1 Department of Ophthalmology, University of Muenster Medical Center, Muenster, Germany; 2 Department of Ophthalmology, Saarland University Medical Center, Homburg/Sarr, Germany; VU University Amsterdam, Netherlands

## Abstract

**Aims:**

To evaluate the relative efficiencies of five Internet-based digital and three paper-based scientific surveys and to estimate the costs for different-sized cohorts.

**Methods:**

Invitations to participate in a survey were distributed via e-mail to employees of two university hospitals (E_1_ and E_2_) and to members of a medical association (E_3_), as a link placed in a special text on the municipal homepage regularly read by the administrative employees of two cities (H_1_ and H_2_), and paper-based to workers at an automobile enterprise (P_1_) and college (P_2_) and senior (P_3_) students. The main parameters analyzed included the numbers of invited and actual participants, and the time and cost to complete the survey. Statistical analysis was descriptive, except for the Kruskal-Wallis-H-test, which was used to compare the three recruitment methods. Cost efficiencies were compared and extrapolated to different-sized cohorts.

**Results:**

The ratios of completely answered questionnaires to distributed questionnaires were between 81.5% (E_1_) and 97.4% (P_2_). Between 6.4% (P_1_) and 57.0% (P_2_) of the invited participants completely answered the questionnaires. The costs per completely answered questionnaire were $0.57–$1.41 (E_1–3_), $1.70 and $0.80 for H_1_ and H_2_, respectively, and $3.36–$4.21 (P_1–3_). Based on our results, electronic surveys with 10, 20, 30, or 42 questions would be estimated to be most cost (and time) efficient if more than 101.6–225.9 (128.2–391.7), 139.8–229.2 (93.8–193.6), 165.8–230.6 (68.7–115.7), or 188.2–231.5 (44.4–72.7) participants were required, respectively.

**Conclusions:**

The study efficiency depended on the technical modalities of the survey methods and engagement of the participants. Depending on our study design, our results suggest that in similar projects that will certainly have more than two to three hundred required participants, the most efficient way of conducting a questionnaire-based survey is likely via the Internet with a digital questionnaire, specifically via a centralized e-mail.

## Introduction

Scientific surveys, such as ophthalmological surveys, have often been conducted using paper-based questionnaires, which must be individually addressed to the potential participants. The collected data must then be inputted manually into software-based statistical programs for analysis. Such procedures are time consuming and may be financially inefficient. A recently suggested alternative in the medical field is digital communication, e.g., using public health tools placed on Internet websites [1], [2] as online self-help interventional tools [3] for clinical reports to improve nursing care [4] or as a potential diagnostic tool for necessary treatments [5], [6].

With regard to electronic and paper case reports, Le Jeannic et al. observed that the former were more advantageous in larger studies and that the total costs of the electronic case reports were lower than those of the paper-based reports. However, they did not suggest a minimal or maximal number that must be included in a cohort to establish a benefit for either method [7].

Electronic and paper-based data collections have been compared in clinical trials and face-to-face interviews with medical professionals who were collecting and inputting the data into either an electronic or paper-based data collection [8], [9]. This method differs from a survey in which the participants input the data themselves for data collection.

Fritz et al. reported their assessment of a web-based application to document questionnaires regarding patient-reported outcomes [10]. They tested their system on a handheld computer, which was directly given to the participants and revealed to be cost efficient; however, their method differed from that of the present indirectly distributed surveys in that they did not use centralized e-mails or homepage links.

In contrast to Fritz et al., Galliher and coworkers could not demonstrate that electronic data collection was sufficiently more efficient than paper-based data collection in a prospective comparison with 1,140 invited participants, as these participants clearly experienced a greater level of technical difficulty when using the electronic devices [11].

The aim of our study was to retrospectively determine and compare the relative recruitment efficacies of three recruitment methods (a paper-based survey and two Internet-based digital surveys) and to virtually estimate the costs of future surveys for different-sized cohorts depending on these results.

## Methods

The surveys used in this study could be answered anonymously, and participation was voluntary. Thus, consent was assumed by the voluntary choice of participating, and this procedure was approved by the ethical approval board (Ethics committee of the Medical Association of Westphalia-Lippe and of the medical faculty of the Westphalian Wilhelms-University, Muenster, Germany) of the university clinics involved.

### Questionnaire content

The survey questions pertained to the motives for or against becoming a postmortem donor of organs and tissues. The first page presented information on the public need for such a survey and on the estimated time required to completely answer all of the questions. This page did not divulge the fact that completion rates would also be measured. For 42 questions, the answer was indicated by simply selecting one of several presented possibilities, including Likert scales with four or five possible answers. The questions collected data regarding the participant's gender, age, religious affiliation, educational level, nationality, family status, profession, general opinions, and motives regarding the postmortem donation of organs, tissues, and, in particular, the cornea. The survey also assessed whether he or she had already discussed this topic with family members or friends and whether he or she was aware of the laws pertaining to postmortem donation.

### Data collection types

Two types of survey questionnaires were used, and their time and cost efficiencies were calculated. Comparing the paper-based (1) and Internet-based digital questionnaire Enterprise Feedback Suite (EFS) Survey by Unipark (QuestBack GmbH, Hürth, Germany) (2), the latter was disseminated via either a centralized e-mail or link on a homepage, i.e., on the daily start-up side of the work computers of all invited employees).

Six variants of the questionnaire had to be included in all three surveys, which differed regarding one informative sentence concerning organ and tissue donation. These variants included such statements as “corneal donation is possible until 72 hours postmortem” and were used to examine a probable influence on general donation attitude. The distribution of these variants had to be randomized for all three survey types. The data from all three surveys were analyzed using the PASW (Predictive Analysis Software) Statistics 22 software (SPSS, Chicago, IL, USA).

#### Nondigital surveys

1) Nondigital surveys with paper-based questionnaires:

The paper-based questionnaire was prepared using Microsoft Word and comprised five pages. It was distributed to the workers of a German automobile enterprise (P_1_), to college students (P_2_), and to senior university students (P_3_). The questionnaires were randomized in advance by manually mixing them and grouping into three paper packs. These packs were offered to the invited participants for unrestricted use, and it was not possible to distinguish the different questionnaire variants externally.

Concerning the automobile enterprise, one week before distribution, the survey was announced with posters to all 4,613 employees who were invited to a business meeting. The survey was also advertised via loudspeakers during the meeting. Participants were able to complete the questionnaire immediately before, during, or after the meeting at the meeting point, depending on their individual needs and predilections. The business meeting took place 87 km from our hospital. The average time taken to answer all of the questions of this paper-based questionnaire had been statistically analyzed in a pilot study with 300 visitors of our university hospital.

The college students (P_2_), aged 16–18 years, had been orally informed in advance by their teachers about the survey and its use. The questionnaires were distributed during their official lessons in the presence of their teachers, who waited to collect the questionnaires immediately after their completion.

The senior university students (P_3_) were not informed in advance about the survey, but a poster was fixed at the entrance of their university classes. When they entered, they were asked by a member of our university to participate in this survey. If they accepted, the questionnaires were handed out and could be returned either following the lessons or by regular mail.

#### Digital surveys

1) Surveys with questionnaires distributed by centralized e-mails:

An e-mail was addressed with a similar introductory text to all employees of our university hospital (E_1_), to another university hospital in southwest Germany (E_2_, Homburg/Saar), and to members of a professional medical association (E_3_) [12]. The e-mail contained a link to the Internet-based questionnaire. The questionnaires had comparable content as the aforementioned paper-based questionnaires and were made available on the Internet. The questionnaire, which contained 12 pages, comprised 12 mandatory questions. Any missing answers resulted in an electronic response to make the participant aware of the missing response. The individual's progress through the questionnaire was signified as a horizontally extending bar on top of the right-hand side of the monitor. The e-mail invitation was resent after 26 days, and the data collection was closed following the sixth week.

2) Surveys with questionnaires linked to homepage invitations:

An invitation placed on a homepage was used to contact the municipal employees of our city (H_1_) and those of an industrial city in the center of Germany (H_2_, Essen). Comparable to the paper-based version, an introductory text regarding matters of organ and tissue transplantation and donation and of the use of the questionnaire was placed at the top of the municipal homepage that regularly appeared when the computer was started. A link was placed in this text that took the participant directly to the survey and was visible for six complete weeks.

Both electronic surveys (centralized e-mails and homepage link) were randomized automatically with the EFS Survey software.

The electronic and paper-based surveys were compared for their question formats and the possibility of randomization. The surveys were also compared with regard to the demographic data and number of potential and effective participants, the time taken to answer all questions, the number of incompletely and completely answered questionnaires, the point in a survey at which the participant abandoned it in partial completion, the time taken to design and implement the survey, and the efficacy of the survey regarding time and financial costs. Complete answering was defined as having responded to the final question of the questionnaire.

To measure the mean time for completing the paper-based questionnaire, the time required to answer the questionnaire was measured in 30 participants in a pilot study. The necessary time for answering the Internet questionnaires was automatically registered with the Survey (QuestBack GmbH, Hürth, Germany).

Based on our observed ratios of potential participants and completely answered questionnaires, the cost and time for surveys with estimated complete questionnaires were calculated for surveys with 10, 20, 30, or 42 questions of the same format.

### Cost calculations

Regarding electronic surveys, we did not consider the costs to depend on the number of presumed participants; however, “designing a basic questionnaire” (DBQ) and “implementation of the questionnaire in an Internet program” (IBQ) were considered to depend on the “number of questions” (NOQ) in comparison to the “number of questions in our survey” (NOQios). The time to “put the data into PASW” (PDS) was considered invariable, and the costs for licensing were not included. NOQ/NOQios is represented by “F” in the following equations:
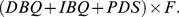



Concerning paper-based surveys, our observed times, costs, and the rate of completely answered questionnaires were taken as comparatives. The process of “designing a basic questionnaire” was regarded as dependent on the number of questions (NOQ). “Paper, copying, and binding” (PCB) and “putting the data into PASW” (PDS) were regarded as dependent on the number of questions and the ratio of “completed questionnaires” (NCQ) in a future survey to the “number of completed questionnaires in our survey” (NCQios). “Distributing and collecting the data” (DC) was regarded as dependent on the number of completed questionnaires.

Costs for transport and materials were not included because they are individual parameters that are location dependent. According to our experience, the time needed to organize both electronic and non-electronic surveys was regarded as similar and thus was also excluded from these calculations.

Costs were calculated with the following formula, where NOQios is the “number of questions in our questionnaire” and NCQios is the “number of completed questionnaires in our survey”: 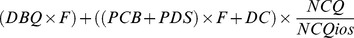.

#### Threshold for cost efficiency

To calculate the number of participants in a paper-based survey that would cost the same as in an electronic survey with an equal number of questions (CEc), we calculated the following: 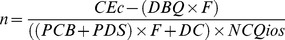.

### Time calculations

The time for performing electronic surveys was considered to be dependent on the number of questions only. Thus, we multiplied the times observed in our survey for DBQ (DBQh) and IBQ (IBQh) according to the number of presumed survey questions (NOQ) in addition to the time necessary to input the data into PASW Statistics 22 (SPSS): 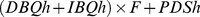.

For paper-based surveys, the time spent “designing a basic questionnaire” (DBQh) was regarded as dependent on the number of questions, with “paper, copying, and binding” (PCBh) and “inputting of the data into PASW” (PDSh) considered dependent on the number of questions, on the size of the target population, and on the presumed completely answered questionnaires. The “distributing and collecting the data” (DCh) was dependent on the number of completely answered questionnaires. Time for transport was not included, as it was considered to be highly variable: 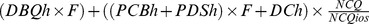.

#### Threshold for time efficiency

To calculate the number of participants in a paper-based survey who would request the same time as in an electronic survey with an “equal number of questions” (CEt), we calculated the following: 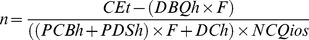.

### Comparison of method efficiencies

The cohorts were grouped into “centralized e-mails”, “homepage links”, and “paper-based questionnaires”, and the Kruskal-Wallis-H-test was used to compare the efficiency rates between the effective and invited participants and between the number of completely answered questionnaires and number of active participants.

## Results

### Demographic data of the participants

Cohort characteristics are listed in Table 1. Differences included gender (e.g., E_1_ and P_1_), age (e.g., P_2_ and P_3_), school education (e.g., P_2_ and E_3_), and the medical or non-medical environment of the participants.

**Table 1:** Demographic parameters of the participants.

	Centralized e-mails			Homepages		Paper-based questionnaires		
	E_1_	E_2_	E_3_	H_1_	H_2_	P_1_	P_2_	P_3_
**Gender**								
Male	29.7%	31.8%	51.0%	42.1%	30.7%	89.5%	48.8%	55.1%
Female	70.3%	68.2%	49.0%	57.9%	69.3%	10.5%	51.2%	44.9%
**Age**								
<18 years	0.1%	0.1%	0.2%	0.0%	0.2%	0.2%	100%	0.0%
18–29 years	20.2%	15.8%	9.4%	5.0%	8.2%	9.4%	0.0%	0.0%
30–49 years	56.6%	56.6%	65.5%	58.9%	51.5%	65.5%	0.0%	0.5%
50–69 years	23.1%	27.0%	22.0%	36.1%	40.2%	22.0%	0.0%	59.5%
>69 years	0.0%	0.6%	2.9%	0.0%	0.0%	2.9%	0.0%	40.0%
**Confession**								
Roman Catholic	58.6%	47.4%	33.3%	63.8%	41.7%	39.1%	56.1%	56.1%
Protestant	26.6%	34.1%	33.3%	63.8%	41.7%	39.1%	56.1%	56.1%
Without confession	14.0%	18.2%	28.7%	19.1%	29.7%	29.7%	14.0%	14.0%
**Scholarship**								
Secondary school	35.4%	35.3%	1.0%	32.3%	33.9%	63.7%	100%	17.2%
University entrance diploma	24.4%	12.2%	2.8%	12.2%	11.6%	17.6%	0.0%	5.3%
University diploma	40.3%	55.5%	96.2%	55.5%	54.5%	18.6%	0.0%	70.9%
**Family status**								
Partnership	64.9%	68.2%	78.0%	74.2%	66.4%	75.3%	12.9%	14.0%
No partnership	35.1%	31.8%	22.0%	25.8%	33.6%	24.7%	87.1%	86.0%
**Medical affiliation**								
Professional medical environment	100.0%	100%	100%	0.0%	0.0%	0.0%	0.0%	37.7%
No professional medical environment	0.0%	0.0%	0.0%	100.0%	100.0%	100.0%	100.0%	62.3%

### Nondigital surveys

#### Nondigital surveys with paper-based questionnaires

It took 8 h to design and formulate the questionnaire in a Word file, which was then used for all paper-based and electronic versions of the survey. Copying and binding 500 transcripts took an additional 4 h for P_1_ and similar times for P_2_ and P_3_, depending on the ability and rates of our students performing these tasks (Table 2). For instance, three students distributed and collected the papers during the employee meeting (P_1_), which took 27 h, and input the resulting data into the statistical software for 24 h. The meeting itself, to which 4,613 employees had been invited, lasted 9 h (Table 2). Our personnel had 500 copies and were able to distribute 303 copies of the questionnaire that were returned; only 294 of the questionnaires were returned completely (Table 3). This corresponds to 97.0% of the number of distributed copies but only 6.4% (294 of 4,613) of the total number of invited employees. The average time taken to answer all of the questions was 9 min and 50 s.

**Table 2:** Time costs of the surveys (^a^ three students working simultaneously).

	Centralized e-mails	Homepages	Paper questionnaires		
Working Process (Abbreviation)	E_1_, E_2_, and E_3_	H_1_, and H_2_	P_1_	P_2_	P_3_
**Designing a basic questionnaire (Word file) (DBQh)**	8 h	8 h	8 h	8 h	8 h
**Implementation of the basic questionnaire in an Internet program (IBQh)**	8 h	8 h	Not necessary	Not necessary	Not necessary
**Paper, Copying and binding (PCBh)**	Not necessary	Not necessary	4 h	9 h	6 h
**Distribution and collecting of the questionnaires (DCh)**	Not necessary	Not necessary	9 h×3^a^ = 27 h	6 h	7 h
**Inputting of the data into PASW 18 (PDSh)**	0.01 h	0.01 h	24 h	28 h	18 h
**Time to completion**	16.0 h	16.0 h	63.0 h	51 h	39.0 h
**Time to survey completion per actual participant**	E_1_: 0.54 min; E_2_: 0.91 min; E_3_: 1.56 min	H1: 1.60 min; H2: 0.88 min	12.47 min	8.97 min	10.88 min

**Table 3:** Number of participants and effective survey responses; ^a^occasionally, computers were professionally used by several employees.

	Centralized e-mails			Homepages		Paper-based questionnaires		
Working Process (Abbreviation)	E_1_	E_2_	E_3_	H_1_	H_2_	P_1_	P_2_	P_3_
**Potential participants**	7,831	5,006	3,887	2,180	8,942	4,613	582	2,108
**Actual participants**	1,771	1,049	614	598	1,092	303	341	215
**Actual participants/potential participants**	22.6%	20.9%	15.8%	27.4%	12.2%	6.6%	58.6%	10.2%
**Provided or distributed questionnaires**	7,831	5,006	3,887	2,180^a^	8,942^a^	500	343	489
**Completely answered questionnaires/opened questionnaires**	1,444 (81.5%)	861 (82.1%)	511 (83.2%)	485 (81.1%)	792 (72.5%)	294 (97.0%)	337 (99.1%)	212 (98.6%)
**Completely answered questionnaires/invited participants**	18.4%	17.2%	13.7%	22.2%	8.9%	6.4%	57.0%	9.6%

Due to the restricted number of personnel, it was not possible to disable communication or interactions between the participants or inhibit the retrograde answering of the questions (i.e., answering of the numbered questions out of order). The total costs of copying the transcripts, and engaging the participants were $1,213.85 (P_1_), $1,151.67 (P_2_), and $854.90 (P_3_), respectively, which is equivalent to $4.13, $3.36, and $4.21 per questionnaire in our surveys (Table 4).

**Table 4:** Financial costs of our surveys and estimated costs based on ^a^$77.8/h, 8 h professional work to establish a digital questionnaire, ^b^$0.05/copy-page, $17.4/h, students working hours (q = questions, p = participants).

	Centralized e-mails/Homepages	Paper questionnaires		
Working Process	E_1–3_, and H_1–2_ (equal results except “costs per participant”)	P_1_	P_2_	P_3_
**Designing a basic questionnaire (Word file) (DBQ)**	33.22 $/10 q; 66.45 $/20 q; 99.67$/30 q; 139.55 $/42 q	33.22 $/10 q; 66.45 $/20 q; 99.67 $/30 q; 139.55 $/42 q	33.22 $/10 q; 66.45 $/20 q; 99.67 $/30 q; 139.55 $/42 q	33.22 $/10 q; 66.45 $/20 q; 99.67 $/30 q; 139.55 $/42 q
**Implementation of the questionnaire in an Internet program^a^ (IBQ)**	148.33 $/10 q; 296.66 $/20 q; 445.00 $/30 q; 623.00 $/42 q	Not necessary	Not necessary	Not necessary
**Paper, copying, and binding^b^ (PBC)**	Not necessary	44.50 $/10 q; 89.00 $/20 q; 133.50 $/30 q; 186.90 $/42 q	100.12 $/10 q; 200.25 $/20 q; 300.37 $/30 q; 420.52 $/42 q	66.75 $/10 q; 133.50 $/20 q; 200.50 $/30 q; 280.35 $/42 q
**License**	62.30 $/10 q; 62.30 $/20 q; 62.30 $/30 q; 62.30 $/42 q	Not necessary	Not necessary	Not necessary
**Distributing and collecting the questionnaires (DC)**	Not necessary	111.86 $/10 q; 223.71 $/20 q; 335.57 $/30 q; 469.80 $/42 q	104.40 $/10 q; 104.40 $/20 q; 104.40 $/30 q; 104.40 $/42 q	121.80 $/10 q; 121.80 $/20 q; 121.80 $/30 q; 121.80 $/42 q
**Putting the data in a statistic program (PASW 18) (PDS)**	1.74 $/10 q; 1.74 $/20 q; 1.74 $/30 q; 1.74 $/42 q	99.24 $/10 q; 198.85 $/20 q; 298.28 $/30 q; 417.60 $/42 q	116.00 $/10 q; 232.00 $/20 q; 348.00 $/30 q; 487.20 $/42 q	74.57 $/10 q; 149.14 $/20 q; 223.71 $/30 q; 313.20 $/42 q
**Total costs**	245.59 $/10 q; 427.15 $/20 q; 608.71 $/30 q; 826.59 $/42 q	288.82 $/10 q, 294 p; 587.01 $/20 q, 294 p; 867.02 $/30 q, 294 p; 1,213.85 $/42 q, 294 p	353.74 $/10 q; 603.10 $/20 q; 852.44 $/30 q; 1,151.67/42 q	296.34 $/10 q; 470.89 $/20 q; 645.68 $/30 q; 854.90 $/42 q
**Costs per participant with completely answered questionnaires**	E_1_: $0.57/42 q; E_2_: $0.84/42 q; E_3_: $1.41/42 q; H_1_: $1.70/42 q; H_2_: $0.80/42 q	$4.13/42 q	$3.36/42 q	$4.21/42 q

### Digital surveys

For digital questionnaires, it was possible to include all questions and question types, as in the paper-based survey. The transfer of the content of the questionnaire Word file to Internet software and subsequent testing before the official start of the electronic survey took 8 h (Table 2). Randomization was achieved using specific software. All of the obtained data could be easily transferred into the statistical software with the survey software employed and took approximately 1.6 min. The average time to complete the questionnaire was 9.17 min. The complete cost for this questionnaire, including the employment of a professional computer specialist for implementation of the basic questionnaire into EFS Survey (8 h), was $826.59 (Table 4).

#### Surveys with questionnaires distributed by centralized e-mails

It took approximately 30 min to organize the centralized distribution of e-mails, which were sent to 7,831 (E_1_) and 5,006 (E_2_) employees of the university hospitals and to 3,887 association members (E_3_). Of these invited, 22.6% (E_1_), 20.9% (E_2_), and 15.8% (E_3_) responded to the questionnaire, and 1,444 (81.5% of the 1,771, E_1_), 861 (82.2% of the 1,049, E_2_), and 511 (83.2% of the 614, E_3_) responded with a fully completed questionnaire (Table 4). Of the 7,831 employees (MU) invited to the questionnaire, 18.4% submitted a completely finished questionnaire (E_2_: 17.2%; E_3_: 13.7%).

The costs per completed questionnaire were $0.57 (E_1_), $0.84 (E_2_), and $1.41 (E_3_) (Table 4).

The first peak response occurred immediately after the first e-mail with 19.1% (E_2_), 26.2% (E_3_), 44.7% (H_2_), and 27.8% (E_1_ and H_1_ together due to digital registration in one data registry) of all of the participants who responded, and the rate gradually decreased thereafter. The response activity increased again following the second reminder e-mail, with 17.0% (E_2_), 12.9% (E_3_), 11.9% (H_2_), and 18.0% (E_1_ and H_1_). The average daily response was 52.1 (E_2_), 33.2 (E_3_), 60.9 (H_2_), and 91.2 (E_1_ and H_1_) participants, which translates to 269 (E_2_), 166 (E_3_), 267.8 (H_2_), and 517 (E_1_ and H_1_) responses per week. Most responses were returned after approximately 10:00 a.m. (E_2_), 7:00 am. (E_3_), 5:00 a.m. (H_2_), and 6:00 a.m. (E1 and H_1_).

#### Surveys with questionnaires linked to homepage invitations

For the centralized homepage questionnaire, it also took approximately 30 min to organize and begin the survey via a municipal homepage. All 2,180 (H_1_) and 8,942 (H_2_) municipal employees were confronted with this homepage at the beginning of their daily work, of which 598 (27.4%, H_1_) and 1,092 (12.2%, H_2_) answered the questionnaire, with 485 (81.1%, H_1_) and 792 (72.5%, H_2_) answering it completely (Table 3).

With regard to H_2_, a response peak was observed immediately after the first homepage invitation (44.7%), which gradually decreased thereafter until the invitation was replaced again at the top of the screen and the responses increased again (11.9%). The average response rate was 60.9 participants per day, corresponding to 267.8 participants per week. The daily response was maximal after 5:00 am.

The costs involved were $1.70 (H_1_) and $0.80 (H_2_) per questionnaire (Table 4).

All three questionnaires, the centralized e-mail, homepage, and paper-based, included the same questions with similar orders, grammars, content, and syntax. All questions types could be completely introduced into the questionnaires. The six questionnaire variants were randomized in both survey types but differently. In the electronic survey, randomization was adjusted for the computer and technically arranged by the EFS Survey, whereas in the paper-based format, such characteristics depended on the individual behaviors of the surveyed employees.

### Estimated costs for electronic and paper-based surveys with different numbers of questions and different cohort sizes

**Figure 1:**
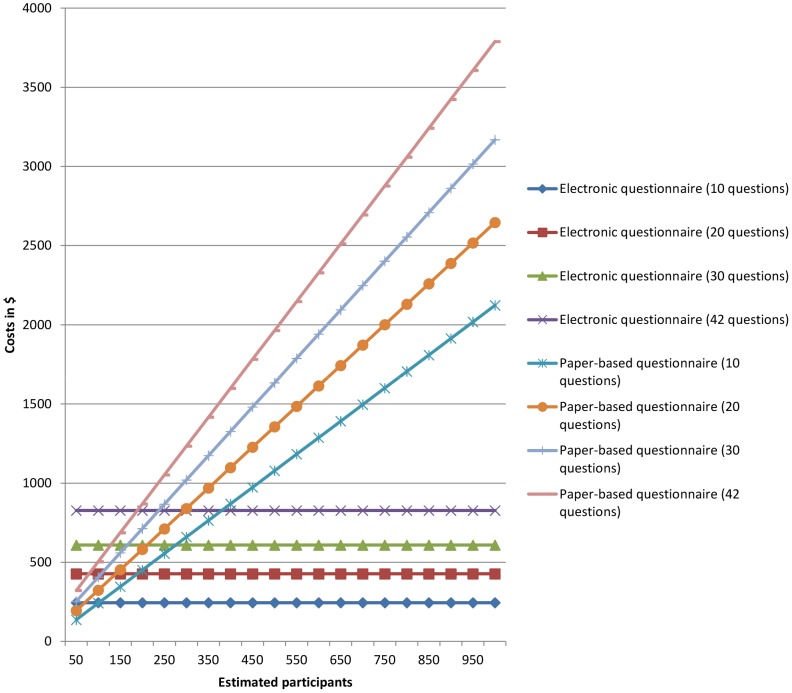
Estimated costs for electronic and paper-based surveys with different cohort sizes and numbers of questions based on the surveys E_1_ (centralized e-mails), H_1_ (homepage link), and P_1_ (paper-based questionnaires).

Total costs for electronic surveys with 10, 20, 30, or 42 questions were calculated to be $245.59, $427.15, $608.71, and $826.59, respectively, independent of the addressed cohort size, whereas the costs for paper-based surveys varied depending on the number of questions and cohort sizes (Fig. 1 and Table 4).

#### Threshold for cost efficiency

Based on our different results, an electronic survey would be more cost efficient than a paper survey if more than 101.60–225.90, 139.81–229.19, 165.80–230.59, or 188.23–231.47 persons are requested to completely answer a questionnaire with 10, 20, 30, or 42 items, respectively (Table 5).

**Table 5:** Estimated costs for electronic and paper-based questionnaires with 10, 20, 30, or 42 questions and different cohort sizes.

	Electronic questionnaires 100, 1,000, or 10,000 participants	Paper-based questionnaires 100, 1,000, or 10,000 participants			Paper-based questionnaire with the same costs as an electronic questionnaire with 10, 20, 30, or 42 questions
Number of questions	Based on our electronic surveys (equal results with: E_1–3_, H_1–2_)	Based on our results with P_1_	Based on our results with P_2_	Based on our results with P_3_	Based on our results with P_1_, P_2_, and P_3_
**10**	245.59 $; 245.59 $; 245.59 $	242.23 $; 2,123.23 $; 20,933.23 $	127.33 $; 973.23 $; 9,433.23 $	155.23 $; 1,286.45 $; 12,233.23 $	n = 101.60; n = 225.90, n = 173.53
**20**	427.15 $; 427.15 $; 427.15 $	324.40 $; 2,646.45 $; 25,866.45 $	223.45 $; 1,636.45 $; 15,766.45 $	254.45 $; 1,946.45 $; 18,866.45 $	n = 139.81; n = 229.19; n = 191.74
**30**	608.71 $; 608.71 $; 608.71 $	406.68 $; 3,169.68; 30,799.68 $	319.68 $; 2,299.68 $; 22,099.69 $	353.68 $; 2,639.68 $; 25,499.68 $	n = 165.80; n = 230.59; n = 200.44
**42**	826.59 $; 826.59 $; 826.59 $	504.55 $; 3,789.55 $; 36,639.55 $	435.55 $; 3,099.55 $; 29,739.55 $	472.55 $; 3,469.55 $; 33,439 $	n = 188.23; n = 231.47; n = 206.49

Costs are calculated with student working hours ($17.4/h), except for the implementation of the questionnaire in an Internet program ($77.8/h, 8 h professional work), and are based on our results according to the different cohorts.

### Estimated time for electronic and paper-based surveys with different numbers of questions and different cohort sizes

**Figure 2:**
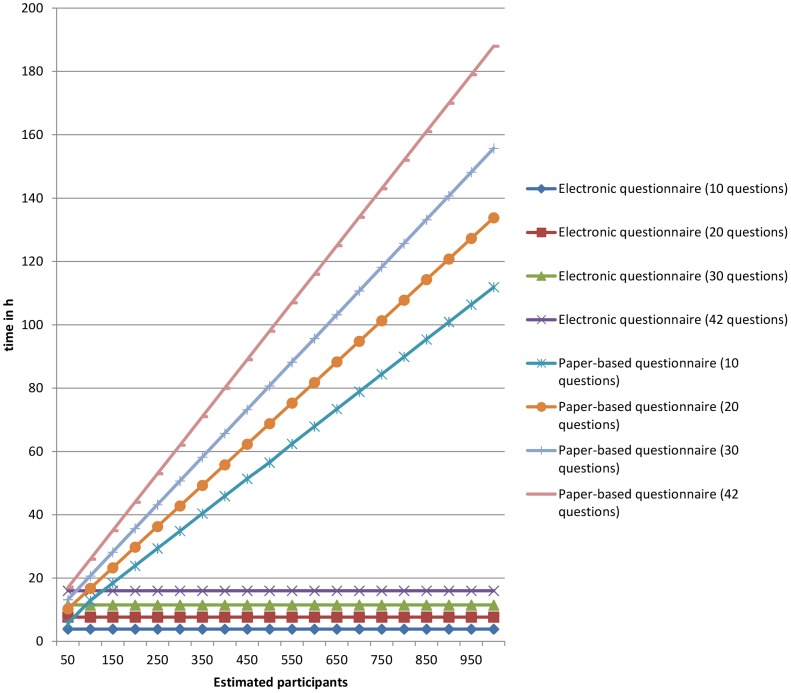
Estimated time required for electronic and paper-based surveys with different cohort sizes and numbers of questions based on the surveys E_1_ (centralized e-mails), H_1_ (homepage link), and P_1_ (paper-based questionnaires).

The calculated times for both electronic surveys, centralized e-mail and homepage, were 3.90, 7.71, 11.52, and 16.01 h (Fig. 2) for questionnaires with 10, 20, 30, and 42 questions, respectively, regardless of the addressed cohort size.

#### Threshold for time efficiency

The calculated times for paper-based surveys were estimated to be different depending on the number of questions on the survey and the cohort sizes.

Based on our results (P_1–3_), an electronic survey would be more time efficient than a paper-based survey if more than 128.18 (P_1_), 227.41 (P_3_), or 391.66 (P_2_) persons are requested to completely answer a questionnaire with 10 items. If questionnaires with 20, 30, or 42 items are to be answered, an electronic survey would be more efficient if more than 93.84, 68.66, or 44.44 (P_1_), 135.55, 87.28, or 52.63 (P_3_), and 193.65, 115.73, or 72.73 (P_2_) persons are required to respond completely, respectively.

### Comparison of method efficiencies

**Figure 3:**
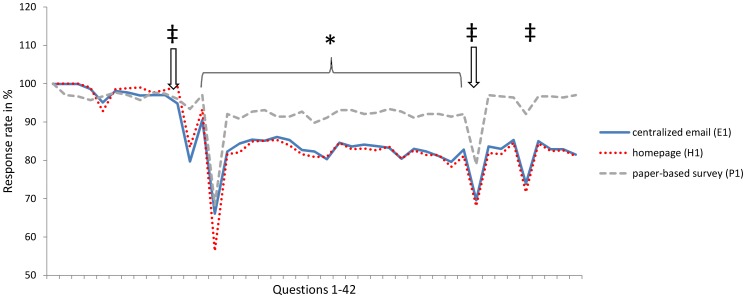
Responses to nonscaled^‡^ and Likert-scaled* questions for the three survey methods (E_1_, H_1_, and P_1_).

The response rates to the questions varied between 59.3% (P_3_), 59.4% (P_2_), 83.2% (P_1_), and 100.0% for the paper-based questionnaires (Fig. 3); 54.6% (E_2_), 66.1% (E_1_), 67.1% (E_3_), and 100%, for the centralized e-mails; and 56.4% (H_1_), 59.4% (H_2_), and 100.00%, for the homepage cohorts. Those questions for which the response called for a Likert score were answered in 97.1–100.0% (P_2_), 96.3–100.0% (P_3_), and 93.4–100.0% (P_1_) of the questionnaires for the paper-based version; in 82.1–100.0% (E_1_), 84.3–100.0% (E_2_), and 85.3–100.0% of the questionnaires for the centralized e-mails; and in 71.3–89.9% (H_1_) and 82.6–87.9% (H_2_) of the questionnaires for the homepage cohorts.

The numbers of invited and active participants are listed in Table 3.

Participants who had started a paper-based questionnaire were more engaged to complete it than participants who respondet to an electronic questionnaire (Table 3). The ratio of completely answered questionnaires to active participants was the most efficient for the use of paper-based invitations (mean rank: 3,462.21), followed by centralized e-mails (2,976.22) and homepage links (2,783.56) (p<0.001).

According to Kruskal-Wallis-H-test, the ratio of active to invited participants was most efficient with centralized e-mails (mean rank: 18,192.14), followed by the paper-based version (17,283.71) and the homepage link (16,729.70) (p<0.001).

## Discussion

We have observed that in our studies, both digital surveys were more time and cost efficient because they were able to address many more participants than the non-digital questionnaire. Because our calculations cannot be generalized, they do not identify a general threshold for cost efficiencies. However, our results encourage us in the future to use electronic surveys if a minimum of 200 participants is requested.

Few studies have evaluated the efficiency of electronic methods of data collection or online research [13]–[18], and concerns regarding the validity of data gathered via Internet-mediated surveys have been reported [19], [20]. To approach different populations for the same survey (i.e., medical professionals, employees of two different university hospitals, nonmedical employees of two large towns, workers at an automobile enterprise, and college and senior university students), we were obliged to choose different questionnaire modalities: electronic and nonelectronic.

Our results are relevant for the topic of our questionnaire (i.e., donation attitudes) but may differ if the topic concerns other subjects, such as health issues. Additional potential bias was observed in the location of the surveys and in the different target populations, including their gender and socioeconomic status, e.g., school education, religion. City employees may be more motivated to participate in a survey performed by and concerning their own hospital, and employees of a hospital may be more engaged if the survey concerns their department. Such influences resulting from different target populations could not be measured in our analysis.

One of our paper-based questionnaires (P_1_) differed from two of our electronic surveys (E_1_ and H_1_) in that the participants of the electronic surveys (E_1_ and H_1_) were offered the possibility of winning a prize, which might have had an influence on their participation and number of answers. In contrast, participants of the other three electronic surveys (E_2–3_ and H_2_) were not given incentives but still presented with relatively high response rates.

In contrast to the electronic survey, participants of the paper questionnaire did not have any comparable reminder, but the surveys were announced via loudspeakers during or via posters before and during the meeting (P_1_, P_3_). Nevertheless, comparatively more participants of the paper-based survey (P_2_) responded to the invitation.

Respondents could not be randomized to conditions because P_1_ employees and college or senior university students did not have centrally organized personal Internet accounts. Due to the large number of E_1–3_ and H_1–2_ employees, it was not possible to distribute a paper questionnaire to all of them. These differences reduced the comparability and external validity of our results.

However, in our opinion, these differences do not explain the relatively low absolute response rate of our paper-based questionnaires P_1_ and P_3_, which simply seems to result from the practical problem of gathering large cohorts and distributing and collecting the questionnaires in a reasonable time frame.

All of the methods allowed the equal use of uni-, bi-, and multilateral, or Likert-scaled questions, and randomization was generally possible in all three surveys.

The time required to answer all of the questions only slightly differed between the paper-based and digital methods. Responses to the nonscaled and Likert-scaled questions were often comparably answered in all three survey methods.

After the data were input into the statistical software, incorrect data were occasionally observed in both the electronic and nonelectronic surveys. Systematic errors, such as incorrect inputting of missing values, were observed with the electronically inputted data. Such errors could be corrected easily and quickly. Nevertheless, incorrect inputting of the data into a software register, e.g. PASW 22, remains possible in both electronic and paper-based surveys, i.e., technical and human proceedings; no system is absolutely secure. Concerning the commercial electronic method, we were not able to check for systematic but invisible errors, the export of datasets from the Internet portal, or their final construction.

Theoretically, digital and paper-based questionnaires can be answered in different ways. Whereas it was possible to begin answering at the end or middle and to continue irregularly in the paper-based form, this was not possible in the digital forms because the software did not allow nonlinear navigation. In addition, interaction between participants in a digital survey is rather unlikely because most of the participants typically complete their own or take the survey subsequently on the same computer. Individual guidance is similarly possible with paper-based surveys but would require an enormous amount of personal organization with additional associated costs. In our opinion, this variation in guidance with the questionnaire type may cause a scientific bias that can only be analyzed correctly if that guidance is systematically reproducible.

In all three surveys, missing answers were observed mainly for the same questions, which may thus be a result of the questions themselves. Furthermore, there were more frequent breaks in the answering with the electronic surveys than with the paper-based surveys when the Likert scales were presented. We presume that such questions are the most taxing to answer; however, the paper-based participants of P_2_ were more motivated to answer the surveys completely probably due to their direct contact with their teachers.

In contrast to the findings of Koo and Skinner, who reported a disappointing survey response of 0.24% following the delivery of 3,801 e-mails in 2005 [14], our response rate following e-mail contact was more encouraging, similar to that of Chen et al., who recommended e-mails as a correspondence tool between general practitioners and patients [21]. Analogous to Hunter et al. [22] and to Hohwü and coworkers [23], who compared electronic questionnaires with postal invitations or paper-based questionnaires, our digital surveys were more time and cost efficient with regard to the absolute number of responses. Although response rates varied between electronic surveys, they were all relatively efficient compared with the paper-based surveys. The college student cohort differed somewhat in its relatively high efficiency; however, this result could have been due to the psychological motivation exacted by their present teachers.

Despite this observation, we presume that electronic surveys, via centralized e-mails or a homepage link, should be able to motivate equal numbers of participants if comparable cohorts are addressed.

A time-consuming part of the survey process for the paper version was distributing and gathering the questionnaires, in addition to the inputting of the data into the statistical program. Our paper-based survey was performed with students who were not paid but were motivated to work efficiently, as they were limited in the amount of time left before their thesis had to be completed. Therefore, in our opinion, a paper-based survey conducted with survey professionals would have been even more expensive. A comparable survey conducted with scannable formats might have been more time and cost efficient than our “old-fashioned typing” of the paper questionnaires. Unfortunately, we did not have access to such methods, the efficiency of which and comparability to electronic surveys should be investigated in further studies.

We have presented the results from eight cohorts with participants of different origins and different socioeconomic status that, in our opinion, approximate the general characteristics of German population. Nevertheless, because we did not perform a prospective, double-armed controlled study, the presented surveys were not completely comparable methodically. Different cohorts, different scenes, and different durations might have affected the numbers of potential and actual participants. Therefore, the results cannot be generalized, although we believe our results reflect the main characteristics of all methods employed. For paper-based surveys, the cost per participant depends more on the respond rate, whereas electronic surveys mainly require a setup cost but still offer the specific advantage of easily stimulating large cohorts several times.

Depending on the intended survey, individual scenes, e.g., different distances to the addressed population, variable characteristics of the cohorts, performance of the used software, and number, arrangement, and intelligibility of the questions, might influence the effectiveness, time, and cost of the survey, which would then differ from our experiences and estimations. It might be more useful to prospectively perform such investigations and aim to approach more comparable cohorts. Nevertheless, the most time- and cost-consuming factors include distributing and gathering the questionnaires; in our opinion, these factors support our observations and calculations.

The results of this study indicated that depending on the complexity of the questionnaire, electronic addressing was more efficient with respect to workflow, responsiveness, time and financial costs for populations of 300 or more participants. On the other hand, the non-electronic survey was more suitable for smaller numbers of potential participants because they could be contacted in person. Although our survey questions pertained to motives for or against being a postmortem donor and tissue donation, we presume that our observations are not restricted to such topics. In our opinion, the presented results are also relevant for public health sciences, which might be able to better support public health improvements. Our estimated calculations might help to organize prospective surveys, especially if general survey characteristics, such as questionnaire items or topics, are already known.
